# Correction to “Loss of Polarity Protein Par3, via Transcription Factor Snail, Promotes Bladder Cancer Metastasis”

**DOI:** 10.1111/cas.70073

**Published:** 2025-04-01

Wang S, Cai J, Zhang S, et al. Loss of Polarity Protein Par3, via Transcription Factor Snail, Promotes Bladder Cancer Metastasis. *Cancer Science* 2021;112:2625–2641, https://doi.org/10.1111/cas.14920.

Some figures in the above article are incorrect.

The corrected Figures 2C, 2E, 5B, 5C, 5D, 5E, 6F and 6G are as follows:

**FIGURE 2**

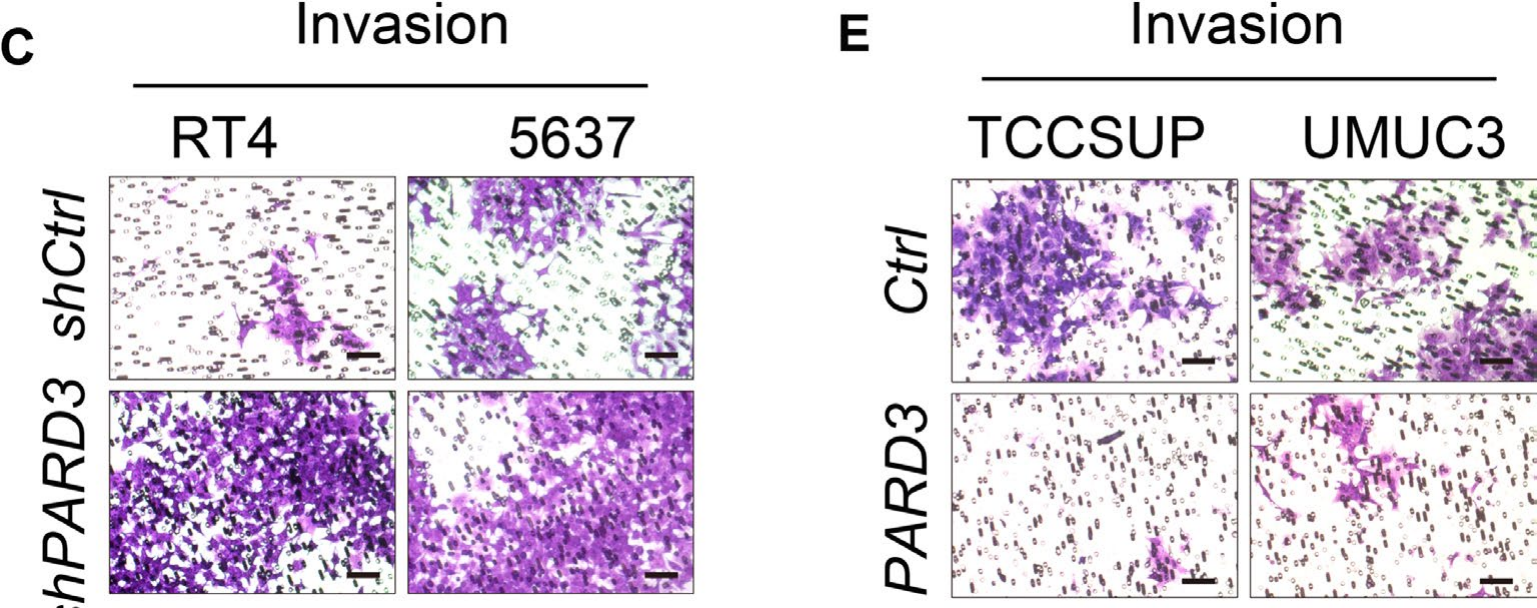

**FIGURE 5**

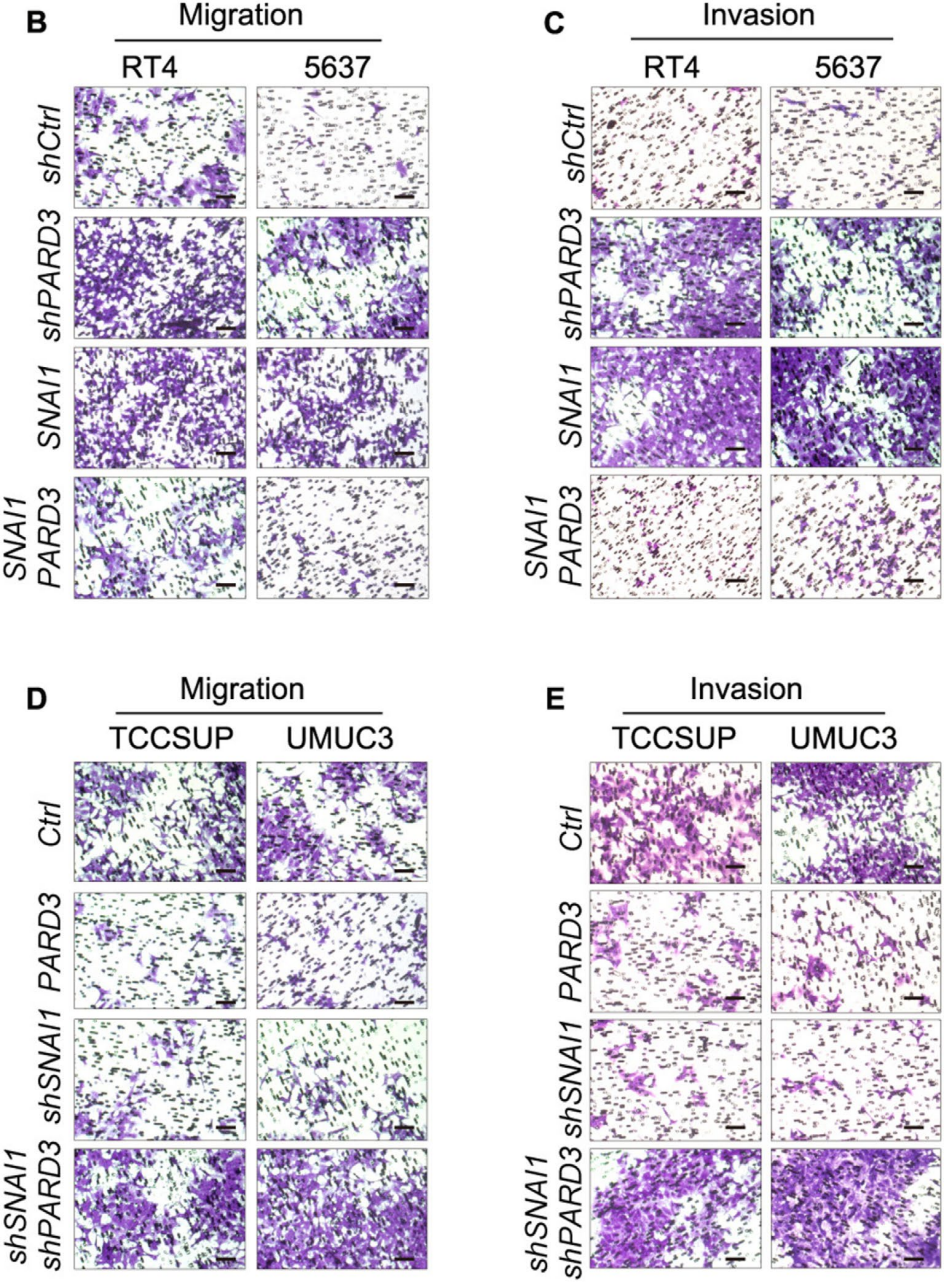

**FIGURE 6**

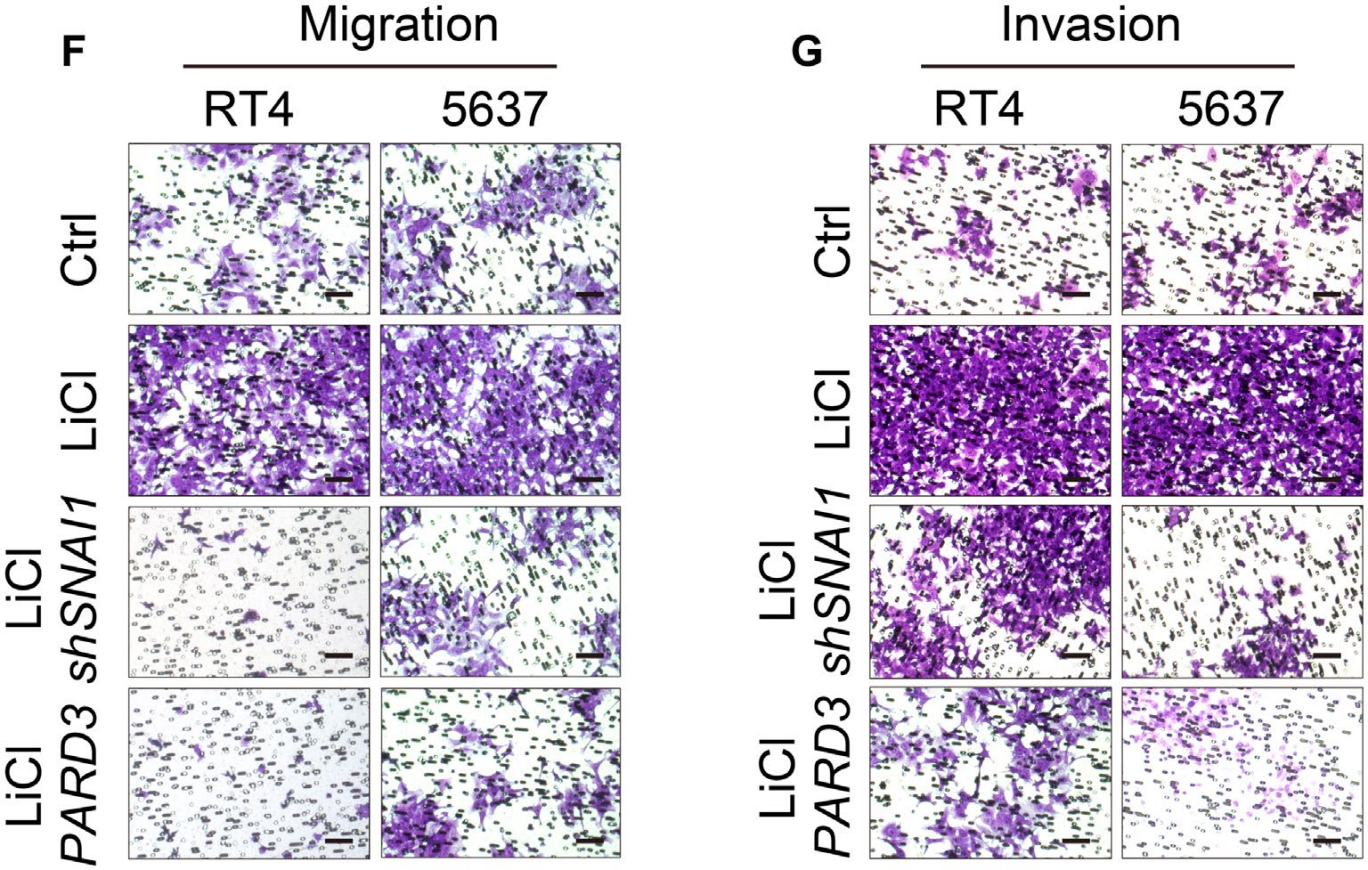

We apologize for this error.

